# The association between physical and mental chronic conditions and napping

**DOI:** 10.1038/s41598-018-37355-3

**Published:** 2019-02-11

**Authors:** Damien Léger, Marion J. Torres, Virginie Bayon, Serge Hercberg, Pilar Galan, Mounir Chennaoui, Valentina A. Andreeva

**Affiliations:** 1 Université Paris Descartes, Sorbonne Paris Cité, EA 7330 VIFASOM, F-75004 Paris, France; 2 APHP Hôtel Dieu, HUPC, Centre du Sommeil et de la Vigilance de l’Hôtel Dieu, F-75004 Paris, France; 3 Université Paris 13, Equipe de Recherche en Epidémiologie Nutritionnelle (EREN), Centre de Recherche en Epidémiologie et Statistiques, Inserm U1153, Inra U1125, Cnam, COMUE Sorbonne Paris Cité, F-93017 Bobigny, France; 4 Institut de Recherche Biomédicale des Armées (IRBA), Unité Fatigue Vigilance, F-91220 Brétigny, France; 5 Département de Santé Publique, Hôpital Avicenne, F-93017 Bobigny, France

## Abstract

The objectives of this study were to assess the associations among various physical and mental chronic conditions and napping. A cross-sectional epidemiological survey was proposed within the NutriNet-Santé population-based e-cohort launched in France in 2009. Participants were 43,060 French volunteers aged 18 y and over with Internet access. A self-report questionnaire assessing sleep characteristics was administered in 2014. The main outcome (dependent) variable was weekday or weekend napping (yes/no). The main exposure (independent) variables were overweight/obesity, hypertension, diabetes, anxiety and depressive disorders, incident major cardiovascular diseases (myocardial infarction, stroke, unstable angina), and incident cancer (breast and prostate). The associations of interest were investigated with multivariable logistic regression analysis. No significant associations were found between major cardiovascular diseases or breast or prostate cancer and napping. Instead, we found that napping was more common among males (46.1%) than among females 36.9% (p < 0.0001). Individuals who were overweight or obese or had hypertension, diabetes, depression or anxiety disorders had an increased likelihood of napping compared with their healthy peers. The adjusted ORs ranged from 1.14 to 1.28″. In conclusion, most chronic conditions were independently associated with napping. Future longitudinal analyses are needed to elucidate causality.

## Introduction

A good night’s sleep is usually recommended as one of the major requirements for good health^1,2^. Sleep has a crucial role in many somatic, cognitive, and psychological processes, and sleeping well appears to be a health imperative, essential for survival^3–6^. However, about one-fifth of adults around the globe are sleeping too little (less than 6 hours per night), and short sleep is associated with a higher risk of obesity, type 2 diabetes (T2D), cancer, hypertension and road/work accidents^7–15^. In addition to sleeping at night, increasing evidence shows that napping may also have real power to relieve physical fatigue and restore alertness^16^. Napping is therefore highly recommended by public health authorities, especially to avoid/compensate for sleep debt and prevent sleep-related road/work accidents^17,18^.

Napping is a natural behaviour common in many countries. In an international survey comparing sleep habits in adults aged 25 to 55 years in six different countries (USA, Canada, Mexico, United Kingdom, Germany, Japan)^19^, the USA and Japan had the highest percentage of nappers (i.e., 51% reporting having taken a nap in the two weeks prior to the survey), followed by the UK (45%), Germany (44%), Mexico (39%) and Canada (35%). In France, a third of the working population indicate that they take at least one nap during the week^20^.

However, even if napping is extensively used in the general population at different moments in life, it is still not consensually recognized if/how chronic physical and mental diseases may be associated with napping habits, even in those with sleep debt^21–25^. In other words, we do not even know people suffering from chronic diseases are using napping on their own, probably facing the severity of their diseases, by increasing their sleep per 24 hours compared with subjects with no disease. Napping could therefore reflect the severity of some chronic diseases, with individuals needing more sleep to cope with fatigue, pain, and possibly inflammatory processes.

Some recent studies have tried to partially answer these questions and have shown that napping was associated with fewer cardiovascular and metabolic diseases^21–25^. Napping by itself might also restore short-term inflammation markers after sleep deprivation or might decrease pain threshold^26,27^. On the other hand, in elderly women, chronic conditions such as diabetes, depressive symptoms, obesity, prevalent stroke and myocardial infarction, dementia and Parkinson’s disease have been associated with napping^28^, independently of nighttime sleep duration and fragmentation. A recent meta-analysis of seven prospective studies, including one US, four European, and two Chinese cohorts, involving a total of 249,077 participants and 13,237 cases of T2D, has shown a 17% increased risk of T2D in habitual nappers compared with non-nappers (RR = 1.17, 95% CI 1.08–1.27)^29^. A recent cross-sectional survey showed that daytime sleeping was associated with significantly higher fasting glucose and insulin in elderly men, even after adjustment for age, body mass index (BMI), physical activity and social class^30^.

To our knowledge, a specific interview on napping and sleep has never been administered to a prospective dataset of subjects to clarify how concise napping characteristics (i.e., the duration and the frequency on weekends and on weekdays) may be associated with chronic diseases. A prospective, cross-sectional study to estimate the odds of napping is needed. Moreover, the association between napping and mental chronic disorders is still quite unexplored. The aim of our study was to survey the napping and sleep traits of a large group men and women whose nutritional and cardiovascular risk factors were assessed, to investigate if different physical or mental chronic diseases were independently associated with napping characteristics.

## Method

### Study population

#### NutriNet-Santé e-cohort

In 2009, the prospective NutriNet-Santé e-cohort was created in France based on an exclusively online survey with a secure Website (www.etude-nutrinet-sante.fr)^31^. Subjects aged ≥18 y were recruited both via Internet access (e.g., website advertising) and via more traditional or multimedia campaigns. It is mandatory for enrolment to give both informed consent and an electronic signature. The NutriNet-Santé prospective cohort was approved by the ethics committee of the French Institute for Health and Medical Research and by the National Commission on Informatics and Liberty. When they were enrolled in the survey, subjects had to systematically fill in a five-part reference questionnaire about socio-demographics and lifestyle, health status, physical activity, anthropometrics, and diet. All participants of the survey regularly received proposals to participate to cross-sectional studies, specifically on our topic of sleep habits. all methods were performed in accordance with the relevant guidelines and regulations.

#### Assessing Napping

All participants of the cohort were requested to complete a self-report questionnaire on sleep characteristics between February and July 2014. It was completed by 57,105 participants on a voluntary basis. In the present analysis, we specifically differentiated the variable (Yes/No) for weekday or week-end napping, which was our main outcome.

#### Chronic disease assessment

The main exposure (independent) variables in the present study were overweight/obesity, hypertension, diabetes (type 1 or type 2), anxiety and depressive disorders, incident major cardiovascular diseases (CVD) (myocardial infarction, stroke, unstable angina), and incident cancer (breast and prostate). Overweight/obesity was estimated via the height and weight self-reported on the sleep questionnaire. Hypertension, diabetes, anxiety and depressive disorders were self-reported at baseline and annually thereafter. Composite variables accounting for prevalent and incident cases of these disorders were calculated. Major CVD and cancer were also self-reported at baseline and annually thereafter. For the present analysis, we used only incident events regarding these diseases.

#### Covariates

As previously reported, the five-part reference questionnaire included socio-demographic, lifestyle (including physical activity, smoking and alcohol use) and health characteristics (height, weight, perceived stress, total sleep time (TST), and daytime sleepiness), which were self-reported. BMI, calculated as the weight (in kg) divided by the squared height (in m), was used as a covariate. Sleep logs included in the sleep questionnaire allowed us to calculate TST among weekdays based on the following items: weekday bedtime, time needed to fall asleep, average duration of nighttime awakenings, and typical morning wake-up time^32^. TST (in minutes) was defined by the difference between the hour when the person falls asleep (calculated by adding the bedtime hour and the time needed to fall asleep) and the wake-up hour. From this number was subtracted the average duration of nighttime awakening. Short TST was defined as sleeping ≤360 minutes per night, normal TST (reference) as sleeping between 361 and 480 minutes per night, and long TST as sleeping ≥481 minutes per night. Daytime sleepiness propensity was assessed via the Epworth Sleepiness Scale (ESS) integrated in the sleep questionnaire^33,34^. The ESS consists of 8 items, asking individuals to self-report the likelihood of dozing off in different situations/contexts, with possible responses ranging from 0 (would never doze) to 3 (high chance of dozing). Thus, the total scores can range from 0 to 24, with a higher score indicating increased daytime sleepiness propensity. Daytime excessive sleepiness propensity was defined as ESS score >10. We evaluated leisure-time physical activity with the International Physical Activity Questionnaire - Short Form^35^. Information on work-related stress was obtained from the sleep questionnaire using the item “Please rate from 1 (most annoying) to 10 (least annoying) the following factors which sometimes or often interfere with your sleep: traffic noise, noisy neighbours, telephone ringing, light pollution, work-related stress, work hours, family issues, relationship difficulties, money worries, health problems.” Individuals who gave a score ≤5 to “work-related stress” were coded as “Yes,” whereas those with scores >5 were coded as “No.” Finally, we modelled as a covariate the interval between enrolment and the sleep questionnaire administration.

### Statistical analysis

The main outcome (dependent) variable was napping (Yes/No). Multivariable logistic regression models, providing odds ratios (ORs) and 95% confidence intervals (95% CIs), were made to test the associations of napping vs. not napping with chronic diseases. Separate analyses were performed for each chronic disease (independent variables, as outlined above) and were therefore adjusted for each of the following covariates: age, sex, BMI, employment category, educational level, marital status, smoking, heavy alcohol use (yes/no), TST, daytime sleepiness, work-related stress, leisure-time physical activity, and interval (in years) between the enrolment in the cohort and the sleep questionnaire administration. In supplementary analyses, we also modelled TST as an exposure variable. Finally, we tested for interaction by sex. All tests were two-sided, and p < 0.01 was considered statistically significant due to the large sample size. All analyses were conducted with SAS software (version 9.4, SAS Institute, Inc., Cary, NC, USA).

## Results

### Sample characteristics

In total, 57,105 individuals completed the sleep questionnaire. The following exclusions were made: non-usable questionnaire data (n = 387); discordant data on any of the sleep items (n = 5,321); missing or non-valuable data regarding napping (n = 96), prevalent cancer (except basal cell carcinoma) (n = 238); prevalent or incident disorders of the immune system (multiple sclerosis, rheumatoid arthritis, Crohn’s disease) (n = 575); pregnancy or recent childbirth (past 4 months) (n = 1,229); and missing covariate data (n = 7,673). Thus, we retained 43,060 subjects (10,485 men and 32,575 women).

The socio-demographic and health status characteristics of the subjects are presented in Table 1 by sex. There were significant differences between men and women for most of the variables. Some 32.6% of men and 31.0% of women were considered short sleepers, whereas 18.4% of men and 23.5% of women were categorized as long sleepers (p < 0.0001). Men were significantly more likely to take naps than were women (46.1% vs. 36.9%, p < 0.0001). However, women tended to take longer naps than did men (40.8 ± 28.5 versus 50.0 ± 34.4 minutes).

**Table 1:** Baseline socio-demographic and health status characteristics of study participants (NutriNet-Santé, n = 43,060).

		Men (n = 10485, 24.3%)		Women (n = 32575, 75.7%)		p
		n or mean	% or SD	n or mean	% or SD	
Age (years)		51.1	14.3	44.8	14.2	<0.0001
Age category	18–34 years	1742	16.6	9214	28.3	<0.0001
	35–59 years	4841	46.2	17453	53.6	
	>=60 years	3902	37.2	5908	18.1	
Marital status	couple	8302	79.2	22648	69.5	<0.0001
Professional inactivity	yes	4896	46.7	12926	39.7	<0.0001
Post-secondary education	yes	6727	64.2	21556	66.2	<0.001
Physical activity^a^	low	4455	42.5	10344	31.8	<0.0001
	moderate	3938	37.6	14885	45.7	
	high	2092	20.0	7346	22.6	
Smoking status	never	4432	42.3	17349	53.3	<0.0001
	ex	4765	45.5	10695	32.8	
	smoking	1288	12.3	4531	13.9	
Heavy alcohol use^b^	no	8943	85.3	23651	72.6	<0.0001
Work-related stress hindering sleep	yes	3398	32.4	14300	43.9	<0.0001
BMI (kg/m²)		25.1	4.1	23.6	4.7	<0.0001
BMI categories	Normal	5830	55.6	23291	71.5	<0.0001
	Overweight	3645	34.8	6279	19.3	
	Obese	1010	9.6	3005	9.2	
Excessive daytime sleepiness^f^	yes	383	3,7	1413	4,3	<0,01
Hypertension^c^	yes	2678	25.5	4219	13	<0.0001
Diabetes (type 1 or type 2)^d^	yes	645	6.2	810	2.5	<0.0001
Major cardiovascular disease (myocardial infarction, stroke, unstable angina)	yes	202	1,9	169	0,5	<0,0001
Depression^e^	yes	327	3.1	1832	5.6	<0.0001
Anxiety^e^	yes	668	6.4	4003	12.3	<0.0001
Breast cancer	yes			284	0.9	
Prostate cancer	yes	123	1.2			

Values refer to number (%) except when noted otherwise.

*p*-values obtained from chi-squared tests and Student *t* tests, as appropriate.

SD = standard deviation.

BMI = body mass index.

^a^Assessed with the International Physical Activity Questionnaire-Short Form; scoring followed the established protocol.

^b^Heavy alcohol use was defined as >30 g/d for men and >20 g/d for women.

^c^Prevalent hypertension based on self-report and/or report of antihypertensive drug use.

^d^Prevalent diabetes type 1 or type 2 based on self-report and/or report of anti-diabetic drug use.

^e^Depression and/or anxiety disorders based on self-report.

^f^Excessive daytime sleepiness as assessed with the Epworth Sleepiness Scale, cutoff score > 10.

### Associations between different chronic diseases and napping

The tests for interaction by sex were not statistically significant, so all models were fit in the full sample. The adjusted associations between different chronic diseases and napping are presented in Table 2. Individuals who were overweight or obese or had hypertension, diabetes (type 1 and 2), or depression or anxiety disorders had a significantly increased likelihood of napping compared with individuals without these disorders. The adjusted ORs ranged from 1.14 to 1.28. No significant associations were found between major CVD or breast or prostate cancer and napping.

**Table 2:** Multivariable logistic regression analysis of the association between chronic diseases and napping (NutriNet-Santé, N = 43,060).

	Napping			p
	OR	95% CI		
**Chronic conditions**				
Overweight or obesity	**1.26**	**1.21**	**1.32**	<**0.0001**
Hypertension	**1.14**	**1.08**	**1.21**	<**0.0002**
Major CVD	**0.96**	**0.78**	**1.19**	**0.70**
Diabetes (type 1 or type 2)	**1.28**	**1.15**	**1.43**	<**0.0001**
Depression	**1.24**	**1.14**	**1.36**	<**0.0001**
Anxiety	**1.19**	**1.12**	**1.27**	<**0.0001**
Breast cancer	**1.09**	**0.85**	**1.39**	**0.52**
Prostate cancer	**1.20**	**0.84**	**1.73**	**0.32**
**Sensitivity analysis**				<**0.0001**
**Total Sleep Time**				
Short sleep vs. normal sleep	**1.14**	**1.09**	**1.19**	
Long sleep vs. normal sleep	**0.98**	**0.93**	**1.03**	

Adjustments were made for the following covariates: age, sex, BMI, employment status, educational level, marital status, smoking, total sleep time, heavy alcohol use, work-related stress hindering sleep (yes/no), leisure-time physical activity (low, moderate, vigorous), and interval between enrolment and sleep questionnaire administration.

95% CI = 95% confidence interval.

### Associations between TST categories and napping

In supplementary analyses, we estimated the adjusted associations between short and long TST and napping, and these results are presented in Table 2. Compared with individuals with normal TST, those with short TST were significantly more likely to take naps (OR = 1.14; 95% CI: 1.09–1.19)). No association with napping was found among individuals with long TST.

## Discussion

In this large, cross-sectional study of adults recruited from the general French population, we for the first time found that different chronic diseases were independently associated with napping across sex. Specifically, subjects with overweight, obesity, hypertension, diabetes (type 1 and 2), or depression or anxiety disorders had an increased likelihood of napping compared with individuals without these disorders. Even if previous studies have already shown an association between napping and some of these diseases, we designed the most detailed survey on napping, which has allowed us to find associations in a vast prospective survey of men and women (NutriNet) independently of other classical metabolic and cardiovascular factors.

It should also be noted that a very high prevalence of napping was observed in the sample: 46.1% of men and 36.9% of women. We specifically and for the first time in detail modelled napping on weekdays or weekends to capture the napping behaviour even among professionally active individuals. Moreover, 26.8% of men and 11.0% of women who took naps did so every day. These rates are close to those reported by a study in six countries led by the National Sleep Foundation^19^. We hypothesize that nappers do not take naps hazardously but rather adopt a sort of daily strategy to extend their sleep. We also observed that short sleepers were more likely to take naps than were normal sleepers.

Another important finding of the present study was that various chronic diseases were differentially associated with napping. For instance, individuals with overweight or obesity were 24% more likely to take weekday or weekend naps, and those with prevalent or incident hypertension were 14% more likely. In turn, individuals with incident major CVD or incident breast or prostate cancer were not more likely than their disease-free counterparts to take naps. Napping is not actually systematically proposed to patients, but they adopt it on their own to cope with fatigue. With these data, interventional strategies around napping may be designed to test how napping may be proposed to patients or associated with other behavioural recommendations, such as physical activity or nutrition.

In the context of chronic metabolic and cardiovascular conditions, too short sleep (<6 hours per 24 hours) promotes chronic sleep debt, which is significantly associated with increased overweight, obesity T2D, hypertension and cardiovascular diseases^7–10^. This may be explained by disturbances in biomarkers of weight regulation (growth hormone, leptin, cortisol) which are secreted during sleep and by behavioural factors (low physical activity, binge eating) which are linked to sleepiness and fatigue^36^. It has been hypothesized that napping may have a beneficial effect on these chronic metabolic and cardiovascular conditions by extending sleep time per 24 hours. In a large prospective cohort, taking a midday nap occasionally (defined as once or twice a week or a midday nap <30 min irrespective of frequency) or ≥3 times/week (average duration ≥30 min) was inversely associated with coronary mortality among healthy working men potentially affected by sleep debt^21,22^. This inverse correlation was obtained after controlling for potential confounders, including smoking status, age, BMI, physical activity, education and Mediterranean diet score.

However, napping has also been associated with increased metabolic and cardiovascular risk in several studies. Napping has been associated with diabetes, obesity, and prevalent stroke and myocardial infarction in elderly women, independently of sleep length^28^. A meta-analysis including 249,077 individuals and 13,237 cases of T2D showed a 17% increased risk of T2D when comparing habitual nappers with non-nappers^29^.

In our study, the average nap time was 40.8 ± 28.5 min in men and 50 ± 30.4 min in women. However, no interaction with sex was observed regarding any of the chronic diseases. Across sex, we found an association between metabolic and cardiovascular conditions and napping, independently of TST and age.

We believe that despite many previous important surveys on the associations between napping and some chronic diseases, there is not enough evidence on how they may be mediated by sleep length. Indeed, the distinction of short and long TST and the control for TST in the main models of our survey is one of the strengths of our analysis. Our results corroborate evidence suggesting that subjects with chronic metabolic or CVD conditions nap more frequently than do their healthy peers. It may therefore be speculated that people develop the habit of napping not only to get additional sleep over a 24-hour period but also to cope with poor health and/or fatigue.

Regarding depression and anxiety, the association with napping has unfortunately been rarely explored. To our knowledge, our study is the first to suggest a significantly increased likelihood of napping in subjects with depression and/or anxiety, independently of TST, sex, age and other potential confounders and using a very large sample derived from the general population. The few previously reported associations between depression and napping have been based on elderly samples^37,38^. Anxiety and depression are usually strongly associated with insomnia^39^. The insomnia definition does not include any reference to short sleep but to increased sleep onset latency and waking after sleep onset^40^. Insomnia is also defined by detrimental daytime consequences such as sleepiness, but it is usually thought that persons with insomnia also have difficulties napping^41^. A study using 7-day actigraphy with 313 participants (aged 34–82 years) identified three patterns of sleep behaviour—infrequent nappers with good night-time sleep, frequent nappers with good night-time sleep, and nappers with poor night-time sleep. Nappers with poor night-time sleep had significantly more depressive symptoms (OR = 4.03) and perceived stress scores (OR = 3.23) compared to the other groups^42^. Next, patients with depression and/or anxiety may be treated by sedative drugs, which may increase their ability to nap. In a group of institutionalized elderly individuals in Australia, the use of benzodiazepines was associated with longer daytime napping (OR = 1.77)^43^. Patients with depression may also feel that napping reduced the severity of their symptoms and thus improved their well-being. A small study reported that patients suffering from major depression who were given the opportunity to nap in the afternoon showed a significant improvement in subjective well-being^44^. Patients with depression might experience increased homeostatic sleep pressure. In a polysomnography-based study with 9 depressed women, 8 healthy young women, and 8 healthy older women, Frey *et al*. observed a higher delta sleep electroencephalogram EEG activity during daytime naps of depressed women compared with healthy young volunteers. They concluded that there was a strong evidence for higher homeostatic sleep pressure in young, moderately depressed women, which may explain their tendency to nap^45^.

Similarly, subjects with chronic anxiety may seek the relaxing effect of napping. From a small EEG-based study with 7 subjects, Luo and Inoue concluded that a short daytime nap modulated affect and thus improved post-nap mental states^46^. The significant positive association we found between anxiety and napping extends prior knowledge in this area.

We believe that these first data on napping and mental disorders may help future research in considering napping as a possible indicator associated with anxiety and depression, adopted by the patients themselves to cope with their sleep disorders and more generally with their psychological symptoms.

Finally, we did not find any significant associations between incident breast or prostate cancer and napping. Cancer of the breast or prostate is hormonally mediated and has higher rates in night workers; incidence of breast or prostate cancer has also been attributed to sleep debt and circadian desynchronization^12,13^. We had hypothesized that these cancers would be associated with napping given their correlation with sleep debt and chronic fatigue. However, the relatively small numbers of breast and prostate cancer patients in our study might have prevented the establishment of a significant association.

### Limitations

We acknowledge several limitations of our study. First, we assessed sleep and napping only by self-report questionnaires. The gold standard in the objective assessment of sleep and napping is polysomnography^40^. When individuals report napping, they might be referring only to rest, without actual sleep. Second, the NutriNet Santé cohort does not represent the general population of France; proportionally, it includes more women and individuals with more formal education (Andreeva *et al*., 2015 – ref below). However, the study sample was very large, with substantial qualitative and quantitative data. Finally, the present study was cross-sectional, and it was not possible to explore the potential causal relationships between chronic diseases and napping^47–49^.
